# Data on patterns of initial recurrence after curative surgery for rectal cancer with neoadjuvant therapy

**DOI:** 10.1016/j.dib.2020.106212

**Published:** 2020-08-22

**Authors:** Zhifang Zheng, Xiaojie Wang, Ying Huang, Xingrong Lu, Zhekun Huang, Pan Chi

**Affiliations:** a Department of Colorectal Surgery, Fujian Medical University Union Hospital, No.29 Xinquan Road, Fuzhou 350001, Fujian, China; b Department of General Surgery, Fujian Medical University Union Hospital, Fuzhou, China

**Keywords:** Rectal cancer, Early recurrence, Recurrence patterns, Neoadjuvant chemoradiotherapy

## Abstract

This paper accompanies the paper titled "Defining and predicting early recurrence in patients with locally advanced rectal cancer treated with neoadjuvant chemoradiotherapy" presented by the same authors to the European Journal of Surgical Oncology [1]. The present article describes the relevant clinical data of patterns of initial recurrence after curative surgery for rectal cancer with neoadjuvant therapy. This data was collected from the hospital records, Chinese Population Registration and Health Insurance System.

**Specifications Table**

**Table:** 

**Subject**	Gastroenterology
**Specific subject area**	rectal cancer
**Type of data**	Figure
**How data were acquired**	The data was collected from the hospital records, Chinese Population Registration and Health Insurance System.
**Data format**	Raw
**Parameters for data collection**	Postoperative follow-up data of rectal cancer patients
**Description of data collection**	Retrospectively acquired medical records of patients
**Data source location**	Fujian Medical University Union Hospital, Fuzhou, China
**Data accessibility**	With the article
**Related research article**	Z. Zheng, X. Wang, Y. Huang, X. Lu, Z. Huang, P. Chi. Defining and predicting early recurrence in patients with locally advanced rectal cancer treated with neoadjuvant chemoradiotherapy. European Journal of Surgical Oncology, in press.

**Value of the Data**

• These data demonstrate the patterns of initial recurrence after curative surgery for rectal cancer with neoadjuvant therapy.
• The data will be beneficial to colorectal surgeons and patients with rectal cancer.
• Through these data, the postoperative recurrence patterns of rectal cancer treated with neoadjuvant therapy can be understood, which will help to guide postoperative surveillance.

## 1 Data description

**Fig. 1:**
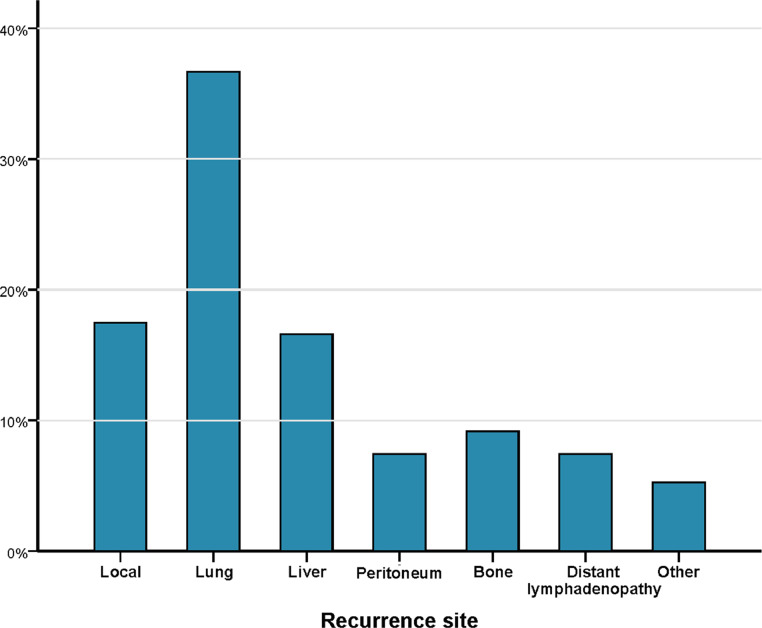
Initial recurrence sites of patients with locally advanced rectal cancer treated with neoadjuvant chemoradiotherapy.

The patterns of initial recurrence after curative surgery for rectal cancer with neoadjuvant therapy (n=167) are presented in Fig. 1. This recurrence data was collected from the hospital records, Chinese Population Registration and Health Insurance System. Raw data of 167 patients with postoperative recurrence are provided in the Supplementary Data.

## 2 Experimental design, materials and methods

From a prospective database, a total of 877 patients with locally advanced rectal cancer who underwent neoadjuvant chemoradiotherapy and radical resection at Fujian Medical University Union Hospital between January 2011 and December 2016 were identified. A surveillance program was provided for all patients. It included follow-up appointments every 3 months for the first 2 years, every 6 months for the next 3 years, and yearly thereafter. Patients were monitored by physical examination, serum carcinoembryonic antigen test, chest computed tomography (CT) scans, and abdominopelvic magnetic resonance imaging (MRI) or CT scans. A colonoscopy was carried out annually. If tumor recurrence was suspected, further studies, such as chest computed tomography, whole-body bone scans, or whole-body positron emission tomography, were performed to determine the site of recurrence. Information on the patients who were lost to follow-up was obtained from the Chinese Population Registration and Health Insurance System. Local recurrence was defined as the regrowth of the tumor within the pelvic cavity. Distant recurrence was defined as any recurrence outside the pelvic cavity. A total of 167 patients had recurrence. The last follow-up date was December 2019 for the surviving patients.

## 3 Ethics statement

The study was approved by the Fujian Medical University Union Hospital Institutional Review Board (No.2020KY093).

## Declaration of Competing Interest

The authors declare that they have no known competing financial interests or personal relationships which have, or could be perceived to have, influenced the work reported in this article.

## Appendix Supplementary materials

The table depicts all patients (n = 167) with postoperative recurrence as raw data. Each line represents one patient. Image, application 1
