# Supplementary material for: Data on patterns of initial recurrence after curative surgery for rectal cancer with neoadjuvant therapy
Source: Data Brief. 2020 Aug 22;32:106212. doi: 10.1016/j.dib.2020.106212 (PMC7452660; doi:10.1016/j.dib.2020.106212)
Supplement: Supplementary file 1 — The table depicts all patients (n = 167) with postoperative recurrence as raw data. Each line represents one patient. [file mmc1.doc]

**Supplementary data:**

| Age | Sex | ypTNM stage | AJCC-TRG | Time of Recurrence (months) | Recurrence site | Adjuvant chemotherapy |
| --- | --- | --- | --- | --- | --- | --- |
| 58 | Male | II | 1 | 9 | Lung | Yes |
| 35 | Female | III | 1 | 5 | Liver | Yes |
| 53 | Female | III | 1 | 20 | Liver | Yes |
| 44 | Male | II | 1 | 48 | Local | No |
| 57 | Male | II | 2 | 48 | Liver | Yes |
| 47 | Male | III | 1 | 26 | Liver | Yes |
| 35 | Female | III | 2 | 43 | Peritoneum | Yes |
| 72 | Male | II | 1 | 42 | Lung | Yes |
| 54 | Male | III | 1 | 12 | Local,Lung | Yes |
| 56 | Female | III | 2 | 7 | Local,Lung,Liver | Yes |
| 65 | Female | pCR | 0 | 72 | Lung | Yes |
| 64 | Male | I | 1 | 70 | Local | Yes |
| 79 | Male | II | 1 | 4 | Local | Yes |
| 64 | Female | I | 0 | 7 | Lung | Yes |
| 40 | Male | III | 1 | 7 | Local | Yes |
| 56 | Male | II | 2 | 26 | Lung | Yes |
| 65 | Male | III | 3 | 6 | Liver | Yes |
| 33 | Female | III | 2 | 19 | Local | Yes |
| 69 | Male | I | 2 | 15 | Peritoneum | Yes |
| 61 | Male | III | 3 | 25 | Lung | Yes |
| 46 | Male | III | 1 | 12 | Local,Peritoneum，Distant lymphadenopathy，Other | Yes |
| 61 | Male | II | 2 | 5 | Lung | Yes |
| 65 | Female | III | 3 | 15 | Lung,Liver,Bone，Peritoneum，Distant lymphadenopathy | Yes |
| 54 | Male | II | 2 | 37 | Lung,Liver | Yes |
| 74 | Male | pCR | 0 | 16 | Liver | Yes |
| 31 | Male | II | 2 | 34 | Local | Yes |
| 49 | Female | III | 1 | 25 | Lung | Yes |
| 59 | Male | II | 2 | 31 | Local,Lung，Distant lymphadenopathy | Yes |
| 63 | Male | pCR | 0 | 54 | Local | Yes |
| 42 | Male | III | 1 | 18 | Lung，Distant lymphadenopathy | Yes |
| 68 | Female | III | 2 | 18 | Liver,Bone，Distant lymphadenopathy | No |
| 64 | Female | III | 2 | 6 | Liver | Yes |
| 58 | Female | II | 2 | 19 | Liver | Yes |
| 66 | Male | III | 2 | 2 | Peritoneum | Yes |
| 42 | Female | III | 1 | 5 | Lung | Yes |
| 37 | Male | III | 2 | 5 | Lung | No |
| 51 | Male | III | 2 | 21 | Lung，Distant lymphadenopathy | Yes |
| 45 | Male | III | 1 | 14 | Lung,Liver | Yes |
| 40 | Female | II | 2 | 27 | Lung，Brain | Yes |
| 51 | Male | III | 2 | 50 | Lung | Yes |
| 45 | Female | III | 1 | 11 | Brain | Yes |
| 66 | Female | III | 1 | 24 | Brain | Yes |
| 79 | Male | II | 2 | 38 | Lung | Yes |
| 48 | Male | pCR | 0 | 37 | Lung | Yes |
| 61 | Male | I | 1 | 23 | Local | Yes |
| 58 | Male | II | 2 | 7 | Liver | Yes |
| 46 | Female | pCR | 0 | 20 | Peritoneum | Yes |
| 30 | Male | III | 2 | 13 | Bone | Yes |
| 56 | Male | I | 2 | 4 | Liver | Yes |
| 55 | Male | I | 1 | 46 | Local | Yes |
| 40 | Male | II | 2 | 6 | Lung,Liver | Yes |
| 54 | Male | I | 1 | 37 | Lung | Yes |
| 66 | Female | II | 2 | 20 | Lung | Yes |
| 58 | Male | III | 1 | 71 | Peritoneum | Yes |
| 46 | Female | III | 2 | 2 | Liver | Yes |
| 57 | Male | III | 2 | 22 | Lung | Yes |
| 49 | Male | III | 1 | 3 | Bone | Yes |
| 47 | Male | I | 2 | 4 | Local | Yes |
| 49 | Female | III | 2 | 49 | Local,Lung,Bone | Yes |
| 51 | Male | I | 1 | 23 | Liver | Yes |
| 48 | Male | III | 2 | 15 | Lung，Distant lymphadenopathy | Yes |
| 45 | Female | II | 1 | 9 | Lung，Distant lymphadenopathy | Yes |
| 53 | Female | III | 1 | 3 | Local,Lung,Bone | Yes |
| 44 | Male | III | 1 | 8 | Bone | Yes |
| 53 | Male | III | 2 | 14 | Lung | Yes |
| 59 | Male | II | 3 | 14 | Lung,Liver | Yes |
| 53 | Male | I | 2 | 68 | Lung | Yes |
| 74 | Male | II | 2 | 35 | Lung | Yes |
| 51 | Male | III | 1 | 9 | Liver | Yes |
| 45 | Male | II | 2 | 11 | Liver | Yes |
| 36 | Female | III | 2 | 35 | Lung，Distant lymphadenopathy | Yes |
| 69 | Male | II | 2 | 16 | Local | Yes |
| 58 | Male | pCR | 0 | 23 | Lung | Yes |
| 78 | Male | III | 2 | 4 | Other | No |
| 76 | Female | III | 3 | 4 | Local,Lung，Peritoneum | Yes |
| 32 | Male | III | 2 | 13 | Liver | Yes |
| 60 | Female | I | 1 | 12 | Lung | Yes |
| 70 | Male | III | 2 | 22 | Lung,Liver | No |
| 59 | Male | III | 3 | 19 | Distant lymphadenopathy,Other | No |
| 57 | Female | III | 2 | 16 | Lung | Yes |
| 60 | Female | III | 2 | 10 | Lung | Yes |
| 60 | Female | I | 1 | 55 | Lung | Yes |
| 40 | Male | II | 3 | 9 | Lung | Yes |
| 60 | Male | II | 1 | 5 | Liver | No |
| 45 | Female | I | 2 | 5 | Liver | Yes |
| 66 | Female | II | 2 | 55 | Bone | Yes |
| 37 | Male | III | 1 | 24 | Local,Peritoneum | Yes |
| 59 | Female | II | 1 | 24 | Local | No |
| 67 | Male | II | 1 | 24 | Liver,Bone | Yes |
| 70 | Male | III | 2 | 19 | Bone | Yes |
| 35 | Male | II | 2 | 19 | Local,Liver，Peritoneum，Distant lymphadenopathy | Yes |
| 52 | Male | II | 1 | 43 | Liver | Yes |
| 51 | Male | III | 2 | 9 | Lung,Bone，Brain | Yes |
| 69 | Female | II | 2 | 5 | Bone | No |
| 33 | Female | III | 2 | 27 | Local，Distant lymphadenopathy | Yes |
| 58 | Female | II | 2 | 19 | Lung | Yes |
| 51 | Male | III | 2 | 11 | Lung，Distant lymphadenopathy | Yes |
| 70 | Male | I | 2 | 12 | Local | Yes |
| 56 | Female | II | 2 | 31 | Local | Yes |
| 68 | Male | II | 2 | 37 | Lung | Yes |
| 73 | Female | II | 2 | 16 | Bone | Yes |
| 52 | Female | III | 2 | 3 | Liver | Yes |
| 64 | Female | pCR | 0 | 39 | Local | Yes |
| 37 | Male | II | 3 | 7 | Local | Yes |
| 42 | Female | III | 1 | 38 | Local | Yes |
| 37 | Male | II | 2 | 11 | Bone | Yes |
| 42 | Male | III | 1 | 17 | Peritoneum | Yes |
| 39 | Male | I | 2 | 19 | Lung | Yes |
| 38 | Male | III | 3 | 12 | Local,Liver | Yes |
| 63 | Male | pCR | 0 | 13 | Lung | Yes |
| 60 | Female | II | 1 | 12 | Lung | Yes |
| 67 | Male | III | 3 | 7 | Lung | Yes |
| 44 | Male | III | 2 | 21 | Lung | Yes |
| 67 | Male | III | 1 | 23 | Lung | Yes |
| 26 | Male | III | 1 | 14 | Local,Liver | Yes |
| 72 | Male | III | 3 | 7 | Lung | Yes |
| 63 | Male | III | 2 | 23 | Local,Lung | No |
| 68 | Female | pCR | 0 | 28 | Lung | No |
| 66 | Male | III | 2 | 24 | Lung,Liver | Yes |
| 50 | Female | II | 2 | 6 | Lung，Peritoneum | Yes |
| 56 | Male | III | 2 | 18 | Lung | Yes |
| 44 | Male | III | 2 | 4 | Bone | No |
| 60 | Male | II | 2 | 6 | Other | Yes |
| 37 | Female | III | 2 | 46 | Local,Lung | Yes |
| 51 | Female | III | 2 | 10 | Lung | Yes |
| 61 | Female | III | 2 | 36 | Lung | Yes |
| 50 | Male | III | 3 | 8 | Peritoneum | Yes |
| 64 | Male | III | 1 | 5 | Lung | Yes |
| 62 | Male | pCR | 0 | 26 | Lung | Yes |
| 42 | Female | I | 2 | 9 | Lung | Yes |
| 69 | Female | III | 2 | 41 | Bone | Yes |
| 38 | Male | III | 2 | 20 | Other | Yes |
| 62 | Female | II | 1 | 21 | Local | Yes |
| 63 | Male | II | 2 | 12 | Local | Yes |
| 50 | Female | II | 1 | 38 | Brain | No |
| 60 | Male | pCR | 0 | 30 | Local,Lung，Distant lymphadenopathy，Other | No |
| 55 | Female | II | 2 | 4 | Local | Yes |
| 66 | Female | III | 1 | 4 | Liver | Yes |
| 47 | Male | III | 1 | 7 | Local，Peritoneum，Distant lymphadenopathy | Yes |
| 62 | Female | III | 1 | 11 | Local，Peritoneum | No |
| 46 | Male | II | 3 | 9 | Local | Yes |
| 52 | Female | III | 2 | 4 | Lung | Yes |
| 47 | Male | III | 2 | 16 | Lung | Yes |
| 65 | Male | II | 2 | 14 | Peritoneum | Yes |
| 61 | Female | III | 3 | 3 | Lung,Liver,Bone | Yes |
| 50 | Male | I | 1 | 6 | Lung | Yes |
| 55 | Male | I | 2 | 24 | Lung,Bone，Distant lymphadenopathy，Brain | Yes |
| 63 | Male | III | 1 | 19 | Lung | No |
| 52 | Male | III | 1 | 21 | Lung | Yes |
| 54 | Female | III | 2 | 6 | Local，Other | Yes |
| 52 | Male | II | 2 | 9 | Local | Yes |
| 59 | Female | pCR | 0 | 3 | Lung | Yes |
| 75 | Male | pCR | 0 | 38 | Lung | No |
| 67 | Male | I | 2 | 3 | Bone，Distant lymphadenopathy | Yes |
| 63 | Male | I | 2 | 3 | Liver | Yes |
| 63 | Male | III | 2 | 13 | Lung,Liver | Yes |
| 64 | Female | II | 2 | 5 | Lung | Yes |
| 50 | Female | II | 3 | 30 | Lung | No |
| 34 | Male | III | 1 | 19 | Peritoneum | Yes |
| 44 | Female | III | 1 | 9 | Lung | Yes |
| 52 | Male | pCR | 0 | 7 | Lung | Yes |
| 53 | Male | III | 2 | 7 | Lung,Liver | Yes |
| 56 | Male | III | 2 | 24 | Lung,Bone | Yes |
| 43 | Male | III | 2 | 7 | Local | Yes |
| 78 | Female | III | 3 | 15 | Bone | Yes |
| 60 | Female | III | 1 | 12 | Lung,Liver | Yes |
| 41 | Male | III | 2 | 10 | Lung | Yes |
